# Rapid Determination of Methanol In Herbaceous Distillates For Their Safety Evaluation by A New Modified Chromotropic Acid Method

**DOI:** 10.22037/ijpr.2019.1100661

**Published:** 2019

**Authors:** Farshid Saadat, Ali Rafizadeh

**Affiliations:** a *Department of Microbiology, School of Medicine, Guilan University of Medical Sciences, Rasht, Iran. *; b *Department of Nursing & Midwifery, Faculty of Nursing & Midwifery, Islamic Azad University, Rasht Branch, Rasht, Iran.*

**Keywords:** Chromotropic Acid, Herbal distillates, Methanol, Chronic methanol poisoning

## Abstract

All sophisticated methods for direct determination of methanol require advanced instruments and high technical knowledge whose preparing them is very expensive in none developed and developing countries. This work reports a simple and efficient qualitative technique for semi determination of methanol content in herbal distillates by a new modified chromotropic acid method. The technique is based on the indirect detection of methanol after its oxidation and transforming to formaldehyde by chromotropic acid (formaldehyde specific color indicator). To measure methanol level in herbal distillates, a water diluted sample was mixed with 50 µL of sulfuric acid and potassium permanganate and after 5 min, followed by addition sodium bi-sulfite, chromotropic acid, and concentrated sulfuric acid in two separated steps and finally, eye comparing with four color standard tubes which gives a range of amount of methanol in the sample. The method has a good precision and accuracy and its Limit of Detection is 25 mgL^-1^. It is particularly suitable for semi quantitative measurement of methanol in herbal distillates not only in the production process quality control of workshops or small companies with no laboratory equipment and adequate financial properties but also the quality check of point of-sale samples from commercial markets. To the best of our knowledge, there isn’t any report about such method.

## Introduction

Human has always used plants and herbs for medicinal purposes. However, the accidental gained knowledge about medicinal plants and their therapeutic uses during long time of human life history has served as a basis for the development of many modern drugs such as aspirin (from willow bark), digoxin (from foxglove), quinine (from cinchona bark), and morphine (from the opium poppy) (1). Recently, the application of plants and herbs products as drugs has increased in modern societies. As, the World Health Organization estimates, about 80% of the population of some Asian countries uses herbal medicine (2). Also, this organization encourages developing countries to use herbal drugs as an alternative to modern systems (3). Therefore, drug regulatory authorities and official public health departments in various countries are actively involved in checking the authentication and safe use of herbal remedies (1).

Herbal distillates is usually a colorless liquid mainly consisting of water and also numbers of very different organic compounds such as diverse drug compounds and essences among which methanol is also found as a unwanted chemical. Because of herbal distillates useful and therapeutic properties, some kinds of them are frequently being used for different purposes in some countries’ (like Iran) food regimen (4-7). Since the methanol content of these products is an important parameter in quality control of herbal distillates production processes, the methods that can efficiently and easily quantify the methanol concentration in these products are highly desired (8-10).

Methanol naturally exists in all plant tissues (leaves, stem, flowers, root and etc.) with more concentrations in green leaves and stems (11-14). This alcohol plays several roles in the plants physiology, as any kind of stress can increase its production in them (11, 15-21). According to American Standard, existence of 120-460 mg L^-1^ (with mean 140 mg L^-1^) methanol in fresh and canned juices is permitted (22). Also, obtained results from several researches confirmed that different amounts of methanol exist in kinds of herbal distillates (8-10).

Methanol is toxic to human and causes poisoning that can be associated with several symptoms (23-25). Very low amounts of ingestion of methanol in the long time can lead to chronic type of methanol poisoning that apparently, unlike sever conditions, the blurred vision leading to blindness is only outcome of it. This type of poisoning due to methanol has recently motivated the Iran contra′s health officials anxieties because, some cases have been reported after drinking large amounts of some kind of herbal distillates during long time (4-7).

Traditionally, methods based on high performance liquid chromatography (HPLC) (26), enzymatic method (21), Fourier transform infrared spectrometric (FT-IR) (27), GC–MS (28) and usually Gas Chromatography (GC) (29, 30) are also used for the determination of methanol in which besides pre-treatment of the samples with HPLC, expensive apparatus are needed in other methods making them inapplicable in common laboratory (31). On the other hand, formaldehyde (HCHO) can be reacted with Chromotropic Acid (CA) in hot concentrated sulfuric acid medium that was adopted as a standard spectrophotometric method for the determination of 0.02–4.00 µgmL^-1^ HCHO (29, 30). So, small amounts of formaldehyde and formaldehyde-releasing compounds can be analyzed by this colorimetric method. Therefore, because of the methanol’s oxidization leads to HCHO formation, it can use CA method for determination of methanol. It is essential to mention that despite the advent of more sophisticated techniques, this method is still widely used because it is simple, sensitive, inexpensive, and very selective (32). This method has been recommended as an official technique by AOAC (Association of Official Analytical Chemists) for measuring of methanol in alcoholic drinks (32, 33). However, CA method requires long operation time for methanol (more than 4 h) and has a painstaking process to treat formic acid which was formed during the oxidation process (34), But, the major drawback of it is the consumption of large volume of hot concentrated sulfuric acid which is potentially hazardous and corrosive (32). Furthermore, this method is only recommended for measurement of methanol in alcoholic drinks (33) and based on it, the application of the main CA method for this purpose in non-alcoholic ones (like herbal distillates) that can lead to gain erroneous results (8-10). In this work, we have successfully developed an alternative effective and specific CA method as an almost micro one with unique properties (no need to heat, severe decreasing of needed concentrated sulfuric acid volume and the other chemicals consumption, very less time consuming, etc.) for the qualitative detection and as contemporaneous, semi determination of methanol content in the herbal distillates based on an old reference method. Therefore, in spite of all mentioned advantages, the functional accuracy of this new modified qualitative chemical CA method is studied compared to GC technique in this paper.

## Experimental

*Reagents and Solutions*

All chemicals used in the experiments, including potassium permanganate, sulfuric acid, sodium hydrogen sulfite and chromotropic acid were analytical grade and purchased from commercial sources and used without further purification. Potassium permanganate, sulfuric acid, sodium hydrogen sulfite and chromotropic acid solutions with 0.1, 0.5, 0.1 and 0.05 molL^-1^ concentrations, respectively were prepared in separated volumetric flasks using de-ionic water. Furthermore, concentrated sulfuric acid was used in the end step of test too. Likewise, two series standard solutions contented 6.25, 12.5, 25, 50, and 100 mgL^-1^ of methanol with ethanol (as internal standard) and 25, 50, 100 and 200 mgL^-1^ of methanol without ethanol were prepared (by serial method) in de-ionic water for using in both GC and proposed chemical methods, respectively. Also, the 35 different herbal distillates samples (*Mentha L., Anethum graveolens L., Alhagi maurorum L., Medicago sativa L., Cichorium intybus L., Salix alba L., Urtica dioica L., Carum carvi L. and Fumaria officinalis L.*) were obtained from the industry and local commercial sources in Iran. For preparing of samples to measure by GC technique, 100µL of the prepared aqueous ethanol solution was added to 10 mL of each sample as internal standard and then, they were injected to GC apparatus directly as triplicate. Whereas, for preparing of samples to be measured by proposed chemical method, one volume of each sample (triplicate) was diluted with equal volume of de-ionic water (a 1:1 ratio) with 1:2 concentration ratio to examine as double.

**Figure 1:**
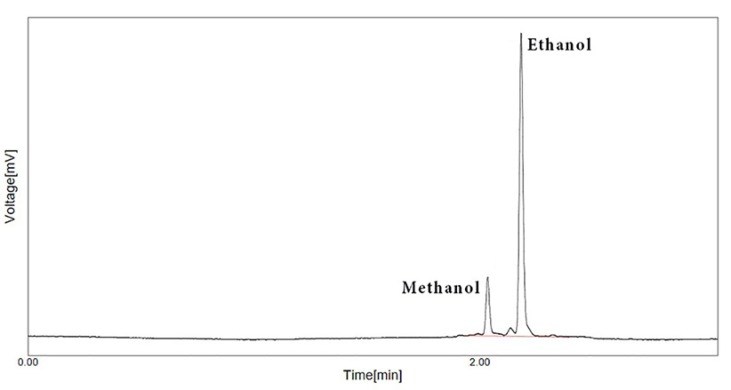
Gas chromatogram of methanol and ethanol in the first standard solution

**Table1:** The average of three times examinations by GC (based on mgL^-1^) and proposed qualitative chemical methods

Company Name of herbal distillate		**A**	**B**	**C**	**D**	**Average of two methods**
***Mentha***	Chemical method	++++ (>200)	++++ (>200)	++++ (>200)	++++ (>200)	(>200)
	GC method	*477*	*294*	*202*	*361*	*333.50*
***Anethum graveolens L.***	Chemical method	++++ (>200)	++++ (>200)	++++ (>200)	++++ (>200)	(>200)
	GC method	*259*	*294*	*112*	*479*	*286.00*
***Alhagi maurorum L.***	Chemical method	++++ (>200)	++++ (>200)	++++ (>200)	++++ (>200)	(>200)
	GC method	*202*	*433*	*397*	*333*	*341.25*
***Medicago sativa*** *** L.***	Chemical method	++++ (>200)	+++ (200)	++++ (>200)	++++ (>200)	(200 - >200)
	GC method	*204*	*149*	*283*	*280*	*229.00*
***Cichorium intybus L.***	Chemical method	++++ (>200)	++++ (>200)	+++ (200)	++++ (>200)	(200 - >200)
	GC method	*205*	*236*	*199*	*275*	*228.75*
***Salix alba*** *** L.***	Chemical method	+ (50)	++ (100)	NS NS	++ (100)	(83.30)
	GC method	*44*	*90*	NS	*86*	*73.33*
***Urtica dioica*** *** L.***	Chemical method	++++ (>200)	++++ (>200)	++++ (>200)	++++ (>200)	(>200)
	GC method	*289*	*268*	*271*	*243*	*267.75*
***Carum carvi L.***	Chemical method	+ (50)	+ + (100)	+ (50)	+ (50)	(62.5)
	GC method	*70*	*95*	*40*	*95*	*75.00*
***Fumaria officinalis*** *** L.***	Chemical method	+ + (100)	+++ (200)	++ (100)	+++ (200)	(150)
	GC method	*102*	*181*	*125*	*225*	*158.25*

*Apparatus*

In this study, a GC apparatus (YL 6100 GC model, South Korea) is used to measure the methanol′s samples. GC system was equipped with a flame ionization detector (FID) and Tr_25_. The length and inner diameter of Si column was 30 m and 0.53 mm, respectively. Helium carrier gas (flow rate = 6 mL.min^-1^) was used for methanol separation. All standards and samples were directly injected (2 µL) to GC system at first incubated at 50 ºC for a minute and then, with 10 ºC/min increased to 80 ^º^C.

*Procedure:*

For performance of tests by proposed chemical method, 0.5mL of each standard and diluted samples were pipetted into separated previous labelled test tubes and then, 50µL of sulfuric acid and potassium permanganate solutions were added into them respectively. After 5 min, the color of mixture was faded by adding 50µL of sodium hydrogen sulfite solution. The test followed by addition of 50µL of chromotropic acid solution and 1mL of concentrated sulfuric acid respectively and five minute were awaited for completion of the reaction. Finally, the results are deduced as below:

1. Negative (absence of methanol or presence of it lower than Limit of Detection (LOD)). The color of mixture remains colorless.

2. Positive (presence of methanol equal or more than LOD). The color of mixture transforms to different intensities of violet. In this case, the methanol content of each sample was estimated by eye comparison of the gained positive results with four standards test tube colors and multiplication of their value in dilution factor (2) to compute the final results.

The chromotropic acid method has got three steps. Briefly, in the first step, the methanol is oxide into formaldehyde by potassium permanganate in acidic medium. Then, in the second step, the hard violet color of solution (due to additional potassium permanganate) is faded by sodium hydrogen sulfite through transforming additional violet mn^7+^ of medium to colorless mn^2+^ to observe the possible positive result. On the final step, the formaldehyde reacts with its specific color indicator (chromotropic acid) in high acidic medium associated with appearance of violet and its intensity is related to samples methanol content.

In the GC method, all prepared standards and samples were directly infused to GC based on before told procedure and then, all gained results were corrected based on internal standard pick. Finally, after computation of results, all attained data about methanol content of herbal distillates by both methods were analysed with SPSS ver. 20 using appropriate tests. One of GC chromatogram has shown in Figure 1. In this Figure, the picks of methanol and ethanol show under the mentioned condition. As it is visible, as for trace methanol concentration in this standard solution, the GC detector can be sufficiently quantified methanol content.

## Results and Discussion

In this study, the LOD of purposed CA method was computed 25 mgL^-1^, which is suitable for a qualitative method. The repeatability of the present method was investigated via triplicate tests of semi determination of methanol in nine herbal distillate samples prepared from some industries and market. Also, to verify the present method, the methanol in herbal distillate samples was determined by both the proposed chemical and GC techniques as a reference method. Moreover, all the examined samples showed different intensities of violet in chemical method whose outcomes were attained via GC technique confirmed them. All obtained results by both methods are shown in table 1.

As it is indicated in table 1, for declaring the differences among the violet intensities in chemical method, the four +, ++, +++, and ++++ signs were used for eye comparison of gained results with four standard solutions containing 25, 50, 100, and 200 mgL^-1 ^of methanol. Based on it, the final computed numerical outcomes of tests have been shown into Parentheses based on mgL^-1^ under the plus signs in table 1 So, as for multiplication of dilution factor (2) for computation of final result, four semi qualitative consequences (50, 100, 200 and >200 mgL^-1^) were practically gained to get compared with GC method results. Also, in the concentrations more than 200 mgL^-1^ of methanol, the recognition of different intensities of violet is impossible, so, all of them are shown as >200 mgL^-1^.

As the results are indicated, all samples have different amounts of methanol that were expected because of essential role of methanol in land plant physiology, and from this point of view; the achieved results in this study is completely similar to the other previously done researches (4-7, 10).

But, the lack of definite average in such cases is caused the comparison of two groups′ results to be difficult. Of course, such condition (limitation in exact measuring or absolute conformity with results gained via quantitative methods) is a common property in all qualitative methods, because rapidness and easiness of application are more importance than accuracy in this type of diagnostic method. However, in such conditions, the application of an appropriate re-dilution of sample (with more than 1:1 ratio) for re-examination of test can be lead to obtain more correct outcome. So, the two achieved results comparison has to be necessarily taken in two separated status as below.

In cases with methanol level with 200 mgL^-1 ^or lower than it, the less difference (8.25 mgL^-1^) between averages of results attained by chemical and GC methods exists in *Fumaria officinalis L.* distillate with 150.00 ± 50.00 and 158.25 ± 48.07 mgL^-1^ concentrations, respectively. Whereas, the highest one (12.50 mgL^-1^) was seen in *Carum carvi L.* distillate with 62.5 ± 21.65 and 75.00 ± 22.64 mgL^-1^ contents, respectively.

But, in cases with 200 - >200 and >200 mgL^-1^ level of methanol in chemical method, the relative conformity of results between each set of two methods outcomes are deductible. So, as for the usual amount of methanol range in different herbal distillates (50 to 200 mgL^-1^) and the nearness of attained results by both used methods together, it can be resulted that the purposed chemical method has suitable precision and accuracy for detection and semi determination of methanol concentration in these products as a rapid and easy qualitative test.

As it is visible in table 1, the range of methanol in given samples are 73.33 and 83.30 to 341.25 and >200 mgL^-1^ in chemical and GC methods, respectively. Unlike samples with methanol more than 200 mgL^-1^ application of a proportional re-dilution for re-examination of test can lead to attain result with more accuracy. Whereas, usage of direct sample (without dilution) for repetition of test in cases less than 50 mgL^-1^ of methanol content is not acceptable. Because, such result is supposed negative and application of direct sample for re-examination of test can lead to gain wrong result (8). In other words, application of diluted sample (at least with 1:1 ratio) is necessary for doing of test in this method.

On the other hand, based on the obtained information from many previously studies (4-8, 10), the amount of methanol in almost all different kinds of herbal distillates (prepared by both industrial and traditional methods in Iran) is usually more than 50 mgL^-1^. The lack of methanol or its existence less than 50 mgL^-1 ^in herbal distillates is probably propounded the cheating and fake origin of product. Therefore, the measuring of such amounts of methanol is not essentially important and furthermore, such amounts of methanol cannot create poisoning. Anyway, in such condition, it can use a quantitative method to determine exact methanol content. Therefore, it seems that the proposed optimum range (50–200 mgL^-1^) for semi determination of methanol in suggested qualitative chemical method is completely suitable.

## Conclusion

According to the explanations and obtained results in this study, it is deductible that all herbaceous distillates have different amounts of methanol. Also, we can develop a reaction based on traditional CA method for semi determination of methanol in these products. Using minimal volume of the reactants instead of their large amounts mentioned in the main method in AOAC can significantly lead to create an easy, safe and rapid method for detection and semi determination of methanol in herbal distillates (a kind of free ethanol drinks) and thus reduces the application problems of the main method. Also, it seems that the present method is simple and has adequate accuracy as a qualitative method for considered aim and also, can be particularly suitable to get used as an easy, versatile, inexpensive, and rapid method for this purpose not only to control production process in small industries with no laboratory equipment, professional knowledge and financial properties at very short time but also the quality check of point-of-sale samples in commercial markets. Also, the gained results by both proposed chemical and GC methods have closely a good conformity together. So, it can use as an applied alternative tool for detection and semi determination of methanol in herbal distillate samples. To the best of our knowledge, there isn’t any report about such method.
